# Risk of cardiovascular disease in cancer survivors: A cohort study of 446,384 New Zealand primary care patients

**DOI:** 10.1002/cam4.6580

**Published:** 2023-09-25

**Authors:** Essa Tawfiq, Romana Pylypchuk, J. Mark Elwood, Mark McKeage, Sue Wells, Vanessa Selak

**Affiliations:** ^1^ School of Population Health University of Auckland Auckland New Zealand; ^2^ School of Medical Sciences University of Auckland Auckland New Zealand

**Keywords:** clinical management, epidemiology, epidemiology and prevention, prognosis, quality of life, survival

## Abstract

**Background:**

Given advances in the management of cancer, it is increasingly important for clinicians to appropriately manage the risk of cardiovascular disease (CVD) among cancer survivors. It is unclear whether CVD risk is increased among cancer survivors overall, and there is inconsistency in evidence to date about CVD incidence and mortality by cancer type.

**Methods:**

Patients aged 30‐74 years entered an open cohort study at the time of first CVD risk assessment, between 2004 and 2018, in primary care in New Zealand. Patients with established CVD or cancer within 2 years prior to study entry were excluded. Cancer diagnosis (1995–2016) was determined from a national cancer registry. Cause‐specific hazard models were used to examine the association between history of cancer and two outcomes: (1) CVD‐related hospitalization and/or death and (2) CVD death.

**Results:**

The study included 446,384 patients, of whom 14,263 (3.2%) were cancer survivors. Risk of CVD hospitalization and/or death was increased among cancer survivors compared with patients without cancer at cohort entry (multivariable‐adjusted hazard ratio, mHR, 1.11, 95% CI 1.05‐1.18), more so for CVD death (1.31, 1.14‐1.52). Risk of CVD hospitalization and/or death was increased in patients with myeloma (2.66, 1.60‐4.42), lung cancer (2.19, 1.48‐3.24) and non‐Hodgkin lymphoma (1.90, 1.42‐2.54), but not for some cancers (e.g., colorectal, 0.87, 0.71‐1.06). Risk of CVD death was increased in several cancer types including melanoma (1.73, 1.25‐2.38) and breast cancer (1.56, 1.16‐2.11).

**Conclusion:**

CVD risk management needs to be prioritized among cancer survivors overall, and particularly in those with myeloma, lung cancer and non‐Hodgkin lymphoma given consistent evidence of increased risk.

## 1 BACKGROUND

With advances in detection and treatment, cancer patients are living longer.^1^, ^2^ For example, in New Zealand (NZ), most people will now survive their cancer, with 66% of all cancer patients surviving at least 5 years after diagnosis.^3^ In order to optimize the health of cancer survivors, their risk of cardiovascular disease (CVD) needs to be understood and adequately managed. CVD, the leading cause of death worldwide,^4^ encompasses a broad range of atherosclerotic conditions including ischemic heart, cerebrovascular and peripheral vascular disease. CVD risk could plausibly be increased among cancer survivors due to shared risk factors (e.g., age, smoking status) as well as cardiotoxic oncologic treatment effects,^5^, ^6^, ^7^ but there is inconsistency and lack of clarity in the evidence to date on the observed risk and mortality of CVD among cancer survivors.

A cancer‐registry based matched cohort study in the United States found no increase in CVD incidence among cancer survivors overall (adjusted incidence rate ratio, aIRR, 1.02, 95% CI 0.99–1.06).^8^ CVD incidence was increased in patients with myeloma (IRR 1.70, 95% CI 1.30–2.21), lung cancer (1.58, 1.30–1.90), non‐Hodgkin lymphoma (NHL, 1.41, 1.20–1.65), and breast cancer (1.13, 1.06–1.22), was reduced in patients with prostate cancer (0.89, 0.84–0.95) and was no different in patients with colon or rectal cancer compared with controls.^8^

A similar cancer‐registry based matched cohort study in the Netherlands also found an increase in the risk of CVD incidence among patients with lung cancer (adjusted hazard ratio, aHR, 1.45, 95% CI 1.04–1.91) and no difference among patients with colorectal cancer.^9^ Unlike the US study, the Dutch study observed no difference in CVD incidence among patients with NHL, breast or prostate cancer compared with controls, and did not assess CVD incidence among melanoma patients.^9^

A UK population‐based matched cohort study assessed the risk of specific CVD outcomes, including coronary artery disease (CAD), among survivors of specific types of cancer.^10^ The UK study similarly found an increase in the risk of CAD among patients with myeloma (aHR 1.87, 95% CI 1.44–2.43), lung cancer (1.42, 1.18–1.72), and NHL (1.30, 1.10–1.54) and no difference in patients with colorectal cancer compared with controls. Unlike the US and Dutch studies, the risk of CAD was increased among patients with prostate cancer (aHR 1.09, 95% CI 1.02–1.17) and was reduced among patients with breast cancer (0.84, 0·77–0.92) in the UK study when compared with controls.^10^ CVD mortality was not evaluated in these studies of cancer survivors.

Given this uncertainty, we sought to determine whether CVD risk (CVD hospitalization and/or death) was increased among cancer survivors overall, and whether risk varied by cancer type, in a large open cohort study of New Zealand (NZ) patients recruited at the time of first CVD risk assessment by their primary care clinician using PREDICT electronic decision support software (the PREDICT cohort).^11^

## 2 METHODS

### 2.1 Participants

PREDICT cohort participants, aged 30–74 years at the date of their first CVD risk assessment using a web‐based platform called PREDICT (index date, between 20 October 2004 and 11 October 2018), were included in this study. Participants were excluded if they already had CVD (or a CVD‐equivalent health condition such as diabetes or overt nephropathy, or diabetes with eGFR<45 mL/min/1.73m^2^, as defined according to the NZ CVD risk prediction equations^12^) or if they were patients within 2 years of their first primary cancer diagnosis, at the index date. The latter group was excluded to minimize any patients still in the acute phase of their cancer treatment from the study, as had been done in a recent study assessing the validity of a CVD risk equation among cancer survivors.^13^ Preceding primary cancer diagnosis (made between 3 January 1995 and 22 June 2016) was determined from the NZ Cancer Registry (NZCR), a legislatively‐mandated registry of all malignancies diagnosed in the country, excluding squamous and basal cell skin cancer.^14^ A primary cancer was defined as a cancer that originated in a body organ or tissue and was neither an extension, a recurrence nor a metastasis of an existing malignancy.^14^ Additionally, a sensitivity analysis was performed by excluding patients who developed cancer during follow‐up, to investigate whether this changes the association with CVD death.

### 2.2 Data sources

Participants' data from the PREDICT cohort study were linked, using an encrypted National Health Identifier (NHI), to four national data collections—NZCR, hospitalization (from public and private hospitals, including coded discharge diagnosis), pharmaceutical dispensing and mortality (including World Health Organization‐classified underlying cause of death), and a regional data collection (Testsafe, for laboratory test results). Every person in NZ who uses health services is assigned an NHI.

### 2.3 Outcomes

Two CVD outcomes were defined: (i) CVD hospitalization and/or death and (ii) CVD death. CVD outcomes were identified using specified ICD‐10‐AM coded diagnoses for ischemic heart disease (including myocardial infarction, angina), ischemic or hemorrhagic cerebrovascular events (including transient ischemic attacks), peripheral vascular disease, congestive heart failure, or other ischemic CVD (but not venous thromboembolism), from hospitalization or mortality data collections.^15^ A CVD death was defined as a death where CVD was the underlying cause of death or if the participant died (irrespective of cause of death) within 28 days of admission for a CVD‐related hospitalization. For the CVD hospitalizations, both secondary and primary diagnoses were used. Patients were followed from the index date until the earliest occurrence of the relevant outcome, non‐CVD death, or the end of follow‐up (2018). The follow‐up was carried out through linkage to the national public and private hospitalization records and mortality datasets covering the entire population present in NZ during the study period.

### 2.4 Predictors

There were two groups of predictors: cancer‐ and CVD‐predictors, in addition to which sex was included where combined (as opposed to sex‐specific) analyses were undertaken. Cancer predictors were: primary cancer at least 2 years prior to the index date (binary) and, among participants with cancer: cancer type (categorical), decade of cancer diagnosis (1995–2005, 2006–2016), and length of time between cancer diagnosis and index date (2–<5 years vs. ≥ 5 years). Only one cancer predictor was used in each model to avoid collinearity. The reference category was always no cancer. The CVD predictors (as used in the NZ CVD risk prediction equations,^12^ all of which were included in all multivariable models) were the following at the index date: age (continuous), self‐identified ethnicity and prioritized in following order (Māori [indigenous population], Pacific, Indian, Chinese or other Asian, and European), socioeconomic deprivation (continuous: NZDep, an area‐based measure), smoking status (categorical: never smoker, ex‐smoker, current smoker), family history of CVD (binary), history of atrial fibrillation (binary), history of diabetes (binary), systolic blood pressure (continuous), ratio of total to high‐density lipoprotein cholesterol (continuous), use of blood pressure‐lowering medication (binary), use of lipid‐lowering medication (binary), and use of antithrombotic medication (binary).

### 2.5 Statistical analysis

Participants were described by all predictors and outcomes, according to sex and cancer status at cohort entry, using numbers and proportions for binary/categorical, and means (standard deviations) for continuous variables. Differences between cancer and non‐cancer patients (for men and women separately) were assessed using the chi‐squared test (binary/categorical) or *t*‐tests (continuous). Because non‐CVD mortality is a competing risk in this study, cause‐specific hazard models were built.^16^ Observations with complete data on all relevant predictors were used. Separate analyses were undertaken for the two outcomes. Two sets of predictors were used in Cox regression models developed for men and women combined, as well as men and women separately: (1) cancer and age only; (2) cancer and all other variables. As noted previously, only one cancer variable was included in each model. Stratified analyses were undertaken whereby analyses were repeated for age (30–60 years, 61–74 years) and follow‐up duration (<5 years, 5+ years) subgroups. All analyses were conducted in Stata (15.1, StataCorp LLC, College Station, TX).

### 2.6 Ethics

The PREDICT study was originally approved by the Northern Region Ethics Committee in 2003 (AKY/03/12/314), with annual approval since 2007 by the National Multi Region Ethics Committee as part of a vascular research programme (2022 EXP 13442).

### 2.7 Funders

The funders of the study (Auckland Medical Research Foundation, Health Research Council of NZ) had no role in study design, data collection, analysis, interpretation, writing of the manuscript or the decision to submit the manuscript for publication.

## 3 RESULTS

**FIGURE 1:**
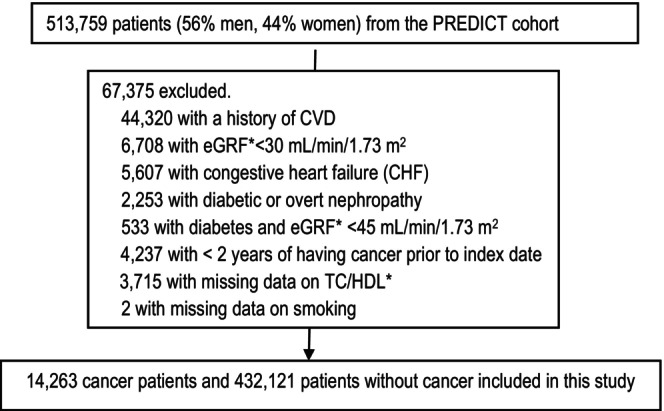
Study flow chart.

Following exclusions, a total of 446,384 patients, of whom 14,263 (3.2%) were cancer survivors, were included in this study (Figure 1).

Cancer patients (mean age 61 and 60 years for men and women, respectively) were older than participants without cancer at the index date (51 and 56 years) (Table 1). There was greater ethnic diversity among participants without cancer (e.g., European participants with cancer: 84% men, 74%, women; without cancer: 54%, 53%). The highest quintile of socioeconomic deprivation, current smoking, family history of CVD and diabetes were more common among people without, compared to those with, cancer. Similarly, people without cancer had higher mean ratio of total to high‐density lipoprotein cholesterol. Cancer patients were more likely to have atrial fibrillation, to be using CVD medication and to have higher mean systolic blood pressure than non‐cancer patients. Follow‐up duration was similar in cancer patients and comparison subjects (men: 5.9 years in both groups; women: 5.8 and 6.0 years, respectively).

**TABLE 1:** Study cohort characteristics, by sex and cancer status at cohort entry.

	Men		Women	
	*N* = 6133 (2.4%)	*N* = 245,751 (97.6%)	*N* = 8130 (4.2%)	*N* = 186,370 (95.8%)
	Cancer	Without cancer	Cancer	Without cancer
Mean age, years (SD)*	61 (9.2)	51 (9.9)	60 (7.8)	56 (8.8)
Self‐identified ethnicity*				
European	5174 (84.4%)	133,433 (54.3%)	5983 (73.6%)	98,315 (52.8%)
Māori	451 (7.3%)	31,153 (12.7%)	870 (10.7%)	26,173 (14.0%)
Pacific	208 (3.4%)	34,665 (14.1%)	571 (7.0%)	26,717 (14.3%)
Indian	93 (1.5%)	21,370 (8.7%)	196 (2.4%)	13,843 (7.4%)
Chinese or other Asian	207 (3.4%)	25,130 (10.2%)	510 (6.3%)	21,322 (11.4%)
NZDep* quintile^a^				
1 (least deprived)	1875 (30.6%)	53,191 (21.6%)	2187 (26.9%)	40,819 (21.9%)
2	1458 (23.8%)	48,852 (19.9%)	1809 (22.3%)	36,751 (19.7%)
3	1088 (17.7%)	44,070 (17.9%)	1516 (18.6%)	33,449 (18.0%)
4	965 (15.7%)	45,633 (18.6%)	1371 (16.9%)	34,331 (18.4%)
5 (most deprived)	747 (12.2%)	54,005 (22.0%)	1247 (15.3%)	41,020 (22.0%)
Smoking*				
Never smoker	4141 (67.5%)	159,449 (64.9%)	5916 (72.8%)	135,401 (72.7%)
Ex‐smoker	1472 (24.0%)	44,974 (18.3%)	1461 (18.0%)	27,694 (14.9%)
Current smoker	520 (8.5%)	41,328 (16.8%)	753 (9.2%)	23,275 (12.5%)
Family history of CVD*	545 (8.9%)	23,387 (9.5%)	930 (11.4%)	21,549 (11.6%)
Atrial fibrillation*	273 (4.5%)	3783 (1.5%)	147 (1.8%)	1843 (1.0%)
Diabetes*	580 (9.5%)	24,318 (9.9%)	801 (9.8%)	22,275 (12.0%)
Mean SBP, mm Hg (SD)*, ^a^	131 (14.5)	129 (14.8)	130 (15.5)	128 (16.1)
Mean TC/HDL (SD)*, ^a^	4.1 (1.2)	4.4 (1.2)	3.6 (1.1)	3.7 (1.1)
Medications at index assessment				
BP‐lowering medication*	1985 (32.4%)	48,717 (19.8%)	2602 (32.0%)	49,479 (26.6%)
Lipid‐lowering medication*	1438 (23.5%)	39,820 (16.2%)	1482 (18.2%)	30,923 (16.6%)
Antithrombotic medication*	1029 (16.8%)	23,781 (9.7%)	980 (12.0%)	18,440 (9.9%)
Decade of cancer diagnosis				
1995–2005	3444 (56.2%)		4727 (58.1%)	
2006–2016	2689 (43.8%)		3403 (41.9%)	
Length between cancer diagnosis date and index date				
2–<3 years	784 (12.8%)		919 (11.3%)	
3–<4 years	716 (11.7%)		802 (9.9%)	
4–<5 years	630 (10.3%)		726 (8.9%)	
≥ 5 years	4003 (65.2%)		5683 (69.9%)	
Length between index date and outcome/end of study				
< 5 years	1890 (30.8%)	92,665 (37.7%)	2803 (34.5%)	69,129 (37.1%)
≥ 5 years	4243 (69.2%)	153,086 (62.3%)	5327 (65.5%)	117,241 (62.9%)
Mean follow‐up time, years (SD)	5.9 (2.6)	5.9 (2.8)	5.8 (2.6)	6.0 (2.8)
Median follow‐up time, (IQR)	5.8 (4.6–7.6)	5.7 (4.2–7.7)	5.7 (4.3–7.4)	5.7 (4.2–7.9)

Abbreviations: SBP, systolic blood pressure; TC/HDL, ratio of total to high density lipoprotein cholesterol.

^a^ Continuous variable. NZDep (New Zealand Index of Socioeconomic Deprivation).

* *p* < 0.05 from the chi‐squared test and *t*‐test conducted between patients with ≥2 years of cancer and patients without cancer.

During follow‐up, more cancer patients (10.7% men, 5.9% women) than non‐cancer patients (6.0% men, 4.6% women) experienced CVD hospitalization and/or death (CVD hospitalization and/or death rate ratio for cancer patients 1.8 for men and 1.3 for women) (Table 2).

**TABLE 2:** Cardiovascular disease (CVD) outcomes, by sex and cancer status at cohort entry.

	Men		Women	
	*N* = 6133 (2.4%)	*N* = 245,751 (97.6%)	*N* = 8130 (4.2%)	*N* = 186,370 (95.8%)
	Cancer	Without cancer	Cancer	Without cancer
Fatal or non‐fatal CVD				
Events, *n*	658 (10.7%)	14,846 (6.0%)	480 (5.9%)	8556 (4.6%)
Person‐years observed	36,381	1,455,844	47,027	1,118,456
Incidence per 1000 (95% CI)	18.1 (16.7–19.5)	10.2 (10.0–10.4)	10.2 (9.3–11.1)	7.6 (7.5–7.8)
Rate ratio	1.8		1.3	
Fatal CVD				
Events, *n*	110 (1.8%)	2050 (0.8%)	90 (1.1%)	1164 (0.6%)
Person‐years observed	38,155	1,502,006	48,279	1,144,155
Incidence per 1000 (95% CI)	2.9 (2.3–3.4)	1.4 (1.3–1.4)	1.9 (1.5–2.2)	1.0 (1.0–1.1)
Rate ratio	2.1		1.9	

In age‐adjusted analyses, the risk of CVD hospitalization and/or death was 7% greater among men and women combined (age‐adjusted hazard ratio, aHR, 1.07, 1.01–1.13) (Table 3). This association of cancer with CVD hospitalization and/or death was marginally augmented after adjustment for all predictors as well as age (multivariable‐adjusted HR, mHR, 1.11, 95% CI 1.05–1.18). This was similar in men and women. The association was broadly consistent whether the cancer diagnosis was made during 1995–2005 or 2006–2016, and whether the time between cancer diagnosis and CVD risk assessment was 2 to 5 or ≥5 years. When stratified by age group (Appendix 1), the association between cancer and CVD hospitalization and/or death only remained statistically significant for people aged 61–74 years (mHR 1.12, 1.05–1.20 and 1.27, 1.08–1.50, respectively) but not for those aged 30–60 years (mHR 1.04, 0.92–1.18 and 1.39, 0.99–1.93, respectively). The association between cancer and CVD hospitalization and/or death was similar and not statistically significant whether follow‐up duration was less or more than 5 years.

**TABLE 3:** Relative rates of cardiovascular disease (fatal or non‐fatal CVD) in survivors of cancer compared with people without cancer, hazard ratio (95% CI).

	Men and women	Men	Women
Cancer status			
Age‐adjusted	1.07* (1.01‐1.13)	1.05 (0.97–1.14)	1.10* (1.00‐1.20)
Multivariable^a^	1.11** (1.05‐1.18)	1.11* (1.03‐1.20)	1.12* (1.02‐1.23)
Years with cancer prior to index date^a^			
2–5 years	1.15** (1.04–1.27)	1.11 (0.97–1.26)	1.23* (1.05‐1.44)
≥ 5 years	1.09* (1.02–1.18)	1.11* (1.01‐1.23)	1.07 (0.96–1.20)
Decade of cancer diagnosis^a^			
1995–2005	1.10* (1.02–1.18)	1.11* (1.01‐1.22)	.08 (0.97–1.21)
2006–2016	1.14* (1.03–1.27)	1.11 (0.97–1.27)	1.21* (1.03‐1.42)
Cancer types^a^			
Breast cancer	1.12 (0.98–1.28)		1.12 (0.98–1.28)
Prostate cancer		1.05 (0.94–1.18)	
Melanoma	1.06 (0.91–1.23)	1.11 (0.93–1.33)	0.98 (0.75–1.27)
Colorectal cancer	0.87 (0.71–1.06)	0.85 (0.65–1.10)	0.90 (0.65–1.23)
Testicular cancer		0.66 (0.35–1.28)	
Non‐Hodgkin lymphoma	1.90** (1.42–2.54)	1.87** (1.26‐2.77)	1.90** (1.23‐2.95)
Kidney cancer	1.69** (1.20–2.38)	1.68* (1.10‐2.55)	1.75 (0.97–3.16)
Leukemia	1.22 (0.79–1.89)	1.45 (0.88–2.41)	0.79 (0.33–1.90)
Cancer of bladder	0.81 (0.49–1.35)	0.81 (0.46–1.42)	0.82 (0.27–2.55)
Lung cancer	2.19 (1.48–3.24)	2.04* (1.16‐3.60)	2.32** (1.35‐4.01)
Stomach cancer	0.71 (0.29–1.70)	0.80 (0.30–2.14)	‐
Myeloma	2.66** (1.60‐4.42)	2.22* (1.11‐4.44)	3.53** (1.68‐7.41)
Hodgkin lymphoma	1.21 (0.50–2.91)	1.27 (0.48–3.39)	‐
Cancer of liver	1.66 (0.69–4.00)	1.15 (0.37–3.56)	‐
Cancer of oral cavity	1.95** (1.36–2.81)	1.99** (1.31‐3.03)	1.79 (0.85–3.76)
Thyroid cancer	0.60 (0.36–1.02)	0.46 (0.17–1.22)	0.69 (0.37–1.29)
Brain cancer	6.66** (3.17–13.98)	‐	‐
Cancer of bone and cartilage	3.48** (1.31–9.29)	‐	‐
Cancer of gallbladder/bile duct	3.44* (1.11–10.66)	‐	‐
Cancer of esophagus	1.63 (0.41–6.54)	‐	‐
Uterine cancer			0.92 (0.65–1.31)
Cancer of cervix			1.14 (0.69–1.89)
Ovarian cancer			1.54 (0.89–2.65)
Vulvovaginal cancer			1.17 (0.49–2.82)

^a^ Adjusted for age, sex, ethnicity, NZDep quintile, smoking, family history of CVD, AF, diabetes, SBP, TC/HDL, BP‐lowering, lipid‐lowering, antithrombotic medications.

* *p* < 0.05

** *p* < 0.01

In terms of CVD deaths, these were also more frequently experienced by cancer patients (1.8% men, 1.1% women) than non‐cancer patients (0.8% men, 0.6% women) (Table 2). The association of cancer with CVD death was more pronounced (age‐adjusted HR, aHR, 1.23, 1.06–1.42; multivariable‐adjusted HR, mHR, 1.31, 1.14–1.52) than its association with the CVD hospitalization and/or death, and was evident for both men and women (mHR 1.24, 1.02–1.51 and 1.44, 1.16–1.79, respectively) (Table 4). As with CVD hospitalization and/or death, the association of cancer with CVD death was broadly consistent whether the cancer diagnosis was made during 1995–2005 or 2006–2016, and whether the time between cancer diagnosis and CVD risk assessment was 2 to 5 or ≥5 year. When stratified by age group (Appendix 1), the association between cancer and CVD death only remained statistically significant for people aged 61–74 years (1.27, 1.08–1.50, respectively) but not for those aged 30–60 years (1.39, 0.99–1.93, respectively), as with CVD hospitalization and/or death. The association between cancer and CVD death was smaller and not statistically significant among those with shorter duration of follow up (mHR 1.14, 0.94–1.38) but, unlike for CVD hospitalization and/or death, was larger and statistically significant among those with longer duration of follow up (mHR 1.40, 1.13–1.72).

**TABLE 4:** Relative rates of cardiovascular disease death (fatal CVD) in survivors of cancer compared with people without cancer, hazard ratio (95% CI).

	Men and women	Men	Women
Age‐adjusted	1.23** (1.06–1.42)	1.12 (0.92–1.36)	1.39** (1.12–1.73)
Multivariable^a^	1.31** (1.14–1.52)	1.24* (1.02–1.51)	1.44** (1.16–1.79)
Years with cancer prior to index date^a^			
2 years–5 years	1.39** (1.10–1.75)	1.17 (0.85–1.61)	1.84** (1.31–2.59)
≥5 years	1.27** (1.06–1.52)	1.28* (1.01–1.63)	1.28 (0.98–1.67)
Decade of cancer diagnosis^a^			
1995–2005	1.35** (1.14–1.60)	1.33* (1.06–1.67)	1.40** (1.09–1.80)
2006–2016	1.22 (0.94–1.59)	1.06 (0.74–1.51)	1.59* (1.07–2.37)
Cancer types^a^			
Breast cancer	1.59** (1.18–2.15)		1.56** (1.16–2.11)
Prostate cancer		0.87 (0.63–1.21)	
Melanoma	1.73** (1.25–2.38)	2.10** (1.46–3.02)	1.07 (0.53–2.15)
Colorectal cancer	0.88 (0.53–1.47)	0.48 (0.20–1.15)	1.56 (0.83–2.91)
Testicular cancer		1.05 (0.26–4.22)	
Non–Hodgkin lymphoma	1.87 (0.84–4.16)	1.66 (0.53–5.14)	2.07 (0.67–6.44)
Kidney cancer	1.41 (0.53–3.75)	1.60 (0.51–4.96)	1.06 (0.15–7.51)
Leukemia	1.74 (0.65–4.64)	2.06 (0.66–6.41)	1.17 (0.16–8.29)
Cancer of bladder	2.25* (1.01–5.01)	1.92 (0.72–5.13)	3.58 (0.89–14.38)
Lung cancer	0.96 (0.24–3.85)	–	2.00 (0.50–8.01)
Cancer of oral cavity	2.17 (0.90–5.22)	2.36** (1.20–6.93)	–
Thyroid cancer	0.89 (0.29–2.76)	0.70 (0.10–4.99)	1.07 (0.27–4.28)
Uterine cancer			0.93 (0.39–2.25)
Cervical cancer			0.52 (0.07–3.72)
Ovarian cancer			2.48 (0.80–7.70)
Vulvovaginal			1.39 (0.20–9.90)
Cancer of stomach	1.75 (0.44–7.02)	2.51 (0.63–10.05)	–
Myeloma	6.17** (2.56–14.85)	5.95** (1.91–18.51)	7.10** (1.77–28.48)
Hodgkin lymphoma	1.77 (0.25–12.60)	2.43 (0.34–17.28)	
Brain cancer	8.36* (1.18–59.43)	–	–
Cancer of gallbladder/bile duct	9.28** (2.96–29.06)	–	–
Cancer of bone and cartilage	13.05** (3.26–52.27)	–	–

^a^ Adjusted for age, sex, ethnicity, NZDep quintile, smoking, family history of CVD, AF, diabetes, SBP, TC/HDL, BP‐lowering, lipid‐lowering, antithrombotic medications.

* *p* < 0.05

** *p* < 0.01.

There were 25,627 patients who developed cancer during the study follow‐up. Associations with CVD death after excluding the 25,627 patients are presented in Appendix 3. Compared with the main analysis estimates presented in Table 3, the hazard ratios have increased slightly for most variables included in models but the confidence intervals mostly overlapped.

Among cancer survivors included in the study, the most common cancer types among men were prostate (37.4%), melanoma (21.8%) and colorectal (10.3%), and for women were breast (47.5%), melanoma (16.2%), and colorectal (7.3%) (see Appendix 2 for the frequency of other cancer types in the cohort). Among men and women, the risk of CVD hospitalization and/or death was increased with myeloma (mHR 2.22, 1.11–4.44 and 3.53, 1.68–7.41 respectively), lung cancer (2.04, 1.16–3.60 and 2.32, 1.35–4.01, respectively) and non‐Hodgkin lymphoma (mHR 1.90, 1.42–2.54 for men and women combined) (Table 3). CVD hospitalization and/or death risk was increased in men with oral cavity cancer (mHR 1.99, 1.31–3.03) but this association was not statistically significant in women with this cancer type (1.79, 0.85–3.76). The risk of CVD death was also increased in both men and women with myeloma (mHR 6.17, 2.56–14.85) as well as the following cancer types: melanoma (1.73, 1.25–2.38), bladder (2.25, 1.01–5.01), brain (8.36, 1.18–59.43), gallbladder/bile duct (9.28, 2.96–29.06) and bone/cartilage (13.05, 3.26–52.27) (Table 4). Among men, the only other cancer types associated with CVD death were oral cavity cancer (mHR 2.36, 1.20–6.93) and melanoma (mHR 2.10, 1.46–3.02) whereas for women only breast cancer was associated with CVD death (mHR 1.56, 1.16–2.11).

## 4 DISCUSSION

In this study of 446,384 patients, cancer survivors (14,263, 3.2%) were more likely than those without cancer to experience CVD hospitalization and/or death (HR 1.11), particularly CVD death (HR 1.31). This association was consistent across follow‐up duration for CVD hospitalization and/or death though was more pronounced for CVD death with longer follow‐up. The increase in CVD risk was even greater in patients with some cancer types, such as myeloma, lung cancer and NHL.

There are notable similarities and differences between our findings and those of the previously described US, Dutch and UK studies.^8^, ^9^, ^10^ CVD risk (non‐fatal or fatal in the US and the UK studies, and non‐fatal only in the Dutch study) has been consistently increased across all the three studies in patients with lung cancer, myeloma (except for the Dutch study in which it was not assessed) and NHL. Patients with NHL in the Dutch study were stratified according to follow up duration (<3 years, 4–13 years) and while no statistically significant increased CVD risk was observed, there was a suggestion of increased risk among those with longer follow‐up duration (aHR 1.46, 95% 0.92–2.32).^9^ There was no increase in CVD risk among colorectal cancer survivors.^8^, ^9^, ^10^ Differences across the studies were evident in terms of breast and prostate cancer. Among breast cancer survivors, our study found an increase in CVD deaths (but not CVD hospitalization and/or death), whereas both the US and the UK studies found an increase in composite CVD, and the Dutch study found no difference in CVD incidence between breast cancer survivors and controls.^8^, ^9^, ^10^ Among prostate cancer survivors, our study found no difference in CVD hospitalization and/or death or CVD death, whereas the US study found a reduction, the Dutch study found no difference and the UK study found an increase in venous thromboembolism.^8^, ^9^, ^10^

Differences in study findings may reflect differences in study design as well as local and temporal factors. The US and Dutch studies were cancer‐registry based matched cohort studies, and the UK study was a population‐based matched cohort study.^8^, ^9^, ^10^ In contrast, our study was based on a cohort of people who had undergone CVD risk assessment in primary care. Criteria for matching varied between those three studies, and matching was not used in our study.^8^, ^9^, ^10^ The Dutch and UK studies included cancer patients who had survived at least 1 year following diagnosis, whereas our and the US studies required survivorship of at least 2 years following diagnosis.^8^, ^9^, ^10^ The US and Dutch studies, as well as our study, assessed risk of a composite CVD outcome, whereas the UK study assessed separate CVD outcomes.^8^, ^9^, ^10^ There were differences in the composite incident CVD outcomes across the three studies that did use these types of outcomes, including whether or not fatal CVD events were included. While all study results were adjusted, variables included in multivariable analysis differed between studies.^8^, ^9^, ^10^

While CVD death wasn't separately assessed in the US, UK, and Dutch studies described above, this outcome was assessed in a different cancer‐registry based US study.^17^ As with our study, that study found an increase in the risk of CVD death among patients with cancer compared with the general US population, after adjustment for age, sex and race.

The observed differences by cancer type (such as increased rates in CVD hospitalization and/or CVD death in patients with myeloma, lung cancer and non‐Hodgkin lymphoma, and increase in CVD deaths in several cancer types including melanoma and breast cancer), may be in part be explained by variations in treatment modalities and time to first diagnosis. In particular, the factors contributing to these differences could be higher survival rates and longer durations at risk of CVD after some cancers (breast/ melanoma); shared risk factors for CVD with some cancers, for example, smoking (lung cancer); cardiotoxic treatment of some cancers, for example, anthracycline chemotherapy (breast cancer, myeloma and non‐Hodgkins lymphoma) or mediastinal radiotherapy (non‐Hodgkins lymphoma).

Limitations of our study are that it was based on a cohort of people enrolled with a primary care practice in which the PREDICT electronic decision support programme was available, and in whom CVD risk assessment was considered appropriate and conducted by a primary care clinician. PREDICT is available in approximately 35%–40% of primary care practices in the Auckland and Northland regions of NZ,^18^ with these two regions representing approximately 35% of the NZ resident population (1.6 million people).^19^ We had excluded patients within 2 years of their first primary cancer diagnosis at index date, to minimize any patients still in the acute phase of their cancer treatment and to facilitate comparison of our results to those of other prominent studies of CVD risk in cancer survivors^8^, ^9^, ^10^ that also excluded subjects with recent cancer diagnosis. Because of this exclusion, the study findings do not apply to people within 2 years of cancer diagnosis. Additionally, we were unable to identify migrants in the study population who could have been diagnosed with a first cancer in another country or those lost to follow‐up due to leaving NZ, although out‐migration would affect both cancer and control groups in this study. According to Statistics New Zealand,^20^ annual out‐migration is less than 1% in all age groups from 55 to 59 upwards, and less than 0.3% above age 70, so under 1% in the age group of our cohort. A recent national analysis found that the mean age of people diagnosed with cancer in NZ between 1 January 1995 and 30 June 2013 was 66 years,^21^ indicating that the cancer survivors in our study were younger (even after taking into account their minimum of 2 years' survival prior to study entry). Other limitations of our study are that we were unable to take cancer treatment, which can be cardiotoxic, into account, and that we did not include venous thromboembolism as an outcome. Lastly, multiple comparisons were performed in this study, however, we have limited the analyses to pre‐specified 10 most common cancer types, thus making sure potentially important findings are not missed.^22^

Advantages of our study were the large size of our cohort, comprehensiveness of linkage to determine preceding cancer status and subsequent CVD hospitalization and/or death due to using both public and private hospitalization as well as mortality data covering all patients who remained in NZ, inclusion of patients with a wide range of cancer types (though we were unable to explore the associations with stage at diagnosis), long follow‐up duration, provision of sex‐specific analyses (as well as combined analyses) and assessment of risk of CVD death, as well as CVD hospitalization and/or death.

The outcomes in our study were established through linkage to national databases of public and private hospitalizations and deaths. The national morbidity and mortality datasets use the WHO‐endorsed ICD‐coding system and are subject to rigorous quality control. While the accuracy of ICD coding for specific diagnoses can be unreliable, the broad definition of CVD as the outcome in our study is likely to counterbalance these possible biases. High sensitivity and positive predictive values for ICD‐coded CVD events in national datasets has been reported by a European study.^23^ Also, a New Zealand study confirmed high level of capture of coronary intervention and associated Acute Coronary Syndromes in the All New Zealand Acute Coronary Syndrome‐QI cardiac register and excellent agreement with national administrative datasets.^24^

Our study indicates that CVD risk management needs to be prioritized among cancer survivors overall, and, consistent with earlier research, particularly those with myeloma, lung cancer and non‐Hodgkin lymphoma in whom there is consistent evidence of increased risk. Further research is needed to better understand CVD risk and mortality in cancer survivors, particularly breast and prostate, given inconsistency in evidence to date, including consideration of the effect of cancer treatment.

## AUTHOR CONTRIBUTIONS

**Essa Tawfiq:** Conceptualization (lead); data curation (equal); formal analysis (lead); funding acquisition (lead); investigation (lead); methodology (equal); writing – original draft (lead); writing – review and editing (equal). **Romana Pylypchuk:** Data curation (equal); formal analysis (equal); investigation (equal); methodology (equal); writing – review and editing (equal). **J. Mark Elwood:** Conceptualization (equal); funding acquisition (equal); investigation (equal); methodology (equal); writing – review and editing (equal). **Mark McKeage:** Conceptualization (equal); formal analysis (equal); investigation (equal); methodology (equal); writing – review and editing (equal). **Sue Wells:** Conceptualization (equal); data curation (equal); investigation (equal); methodology (equal); writing – review and editing (equal). **Vanessa Selak:** Conceptualization (lead); data curation (equal); formal analysis (equal); funding acquisition (equal); investigation (equal); methodology (equal); writing – review and editing (equal).

## CONFLICT OF INTEREST STATEMENT

ET's salary was supported by the Auckland Medical Research Foundation (AMRF) grant, project number 3722609, grant reference 1,120,015. RP's research was supported by the Health Research Council of New Zealand (HRC) grant. JME's research was supported by the AMRF and HRC. MMcK's research was supported by the HRC. SW's research was supported by grants from AMRF, HRC, National Heart Foundation of NZ (NHF) and the Stevenson Foundation. VS's research was supported by grants from the AMRF, HRC, NHF and National Science Challenge (Healthier Lives) (NSCHL).

## Data Availability

All individual participant (deidentified) data, including a data dictionary defining each field, will be made available to university‐based academic researchers if their proposed analyses are approved by the investigators' Data Access Proposal Committee. A proposal must be considered relevant to the original aims of the research, must meet the study's ethics approval criteria, and will require one or more of the study investigators as formal collaborators. A signed data access agreement will be required and the costs of preparing the datasets will need to be covered. There are no set dates for when these data will be made available. Please contact the corresponding author regarding data sharing requests.

## APPENDIX 1 Relative rates of cardiovascular disease (CVD) and CVD death by age category and follow‐up time in survivors of cancer compared with people without cancer, hazard ratio (95% CI)

### 1.1

**Table:**

	Men and women	Men	Women
CVD hospitalization and/or death			
Age 30–60 years			
Age‐adjusted	0.89 (0.79–1.01)	1.0 (0.84–1.18)	0.93 (0.78–1.12)
Multivariable	1.04 (0.92–1.18)	1.12 (0.94–1.32)	0.97 (0.81–1.17)
Age 61–74 years			
Age‐adjusted	1.12** (1.04–1.20)	1.06 (0.97–1.16)	1.16** (1.04–1.29)
Multivariable	1.12** (1.05–1.20)	1.09 (0.99–1.19)	1.17** (1.05–1.30)
Follow‐up time <5 years			
Age‐adjusted	1.06 (0.98–1.14)	1.09 (0.99–1.20)	1.06 (0.95–1.19)
Multivariable analysis	1.06 (0.98–1.14)	1.07 (0.98–1.18)	1.03 (0.92–1.16)
Follow‐up time ≥5 years			
Age‐adjusted	1.01 (0.91–1.12)	0.99 (0.86–1.14)	1.03 (0.88–1.20)
Multivariable	1.05 (0.95–1.16)	1.06 (0.92–1.22)	1.04 (0.89–1.21)
CVD death			
Age 30–60 years			
Age‐adjusted	1.12 (0.81–1.55)	1.24 (0.81–1.93)	1.23 (0.75–2.03)
Multivariable	1.39 (0.99–1.93)	1.49 (0.96–2.29)	1.28 (0.78–2.12)
Age 61–74 years			
Age‐adjusted	1.22* (1.04–1.44)	1.06 (0.85–1.32)	1.43** (1.13–1.82)
Multivariable	1.27** (1.08–1.50)	1.14 (0.91–1.41)	1.48** (1.16–1.88)
Follow‐up time <5 years			
Age‐adjusted	1.13 (0.93–1.39)	1.07 (0.82–1.40)	1.32 (0.97–1.78)
Multivariable	1.14 (0.94–1.38)	1.06 (0.81–1.39)	1.28 (0.95–1.74)
Follow‐up time ≥5 years			
Age‐adjusted	1.30* (1.06–1.61)	1.24 (0.93–1.64)	1.38* (1.01–1.87)
Multivariable	1.40** (1.13–1.72)	1.39* (1.05–1.85)	1.41* (1.04–1.93)

**p* < 0.05

**p* < 0.01.

## APPENDIX 2 Cancer type, by sex, among patients with cancer at cohort entry

### 2.1

**Table:**

	Men *N* = 6133	Women *N* = 8130
Prostate cancer	2294 (37.4%)	
Melanoma	1331 (21.7%)	
Colorectal cancer	639 (10.4%)	
Testicular cancer	314 (5.1%)	
Non‐Hodgkin lymphoma	235 (3.8%)	
Cancer of oral cavity	152 (2.5%)	
Kidney cancer	150 (2.4%)	
Leukemia	135 (2.2%)	
Cancer of bladder	129 (2.1%)	
Thyroid cancer	92 (1.5%)	
Lung cancer	65 (1.1%)	
Cancer of stomach	61 (1.0%)	
Myeloma	59 (1.0%)	
Hodgkin lymphoma	59 (1.0%)	
Brain cancer	32 (0.8%)	
Liver cancer	31 (0.5%)	
Cancer of esophagus	15 (0.4%)	
Cancer of bone and cartilage	13(0.4%)	
Other male genital cancer	9(0.3%)	
Cancer of gallbladder/bile duct	7(0.2%)	
Breast cancer	6(0.2%)	
Other cancers in men	305 (5.0%)	
Breast cancer		3862 (47.5%)
Melanoma		1318 (16.2%)
Colorectal cancer		630 (7.7%)
Uterine cancer		456 (5.6%)
Thyroid cancer		321 (3.9%)
Cancer of cervix		268 (3.3%)
Non‐Hodgkin lymphoma		224 (2.8%)
Ovarian cancer		180 (2.2%)
Kidney cancer		109 (1.3%)
Leukemia		99 (1.2%)
Lung cancer		96 (1.2%)
Cancer of oral cavity		65 (0.8%)
Vulvovaginal cancer		56 (0.7%)
Cancer of bladder		45 (0.6%)
Myeloma		44 (0.5%)
Cancer of stomach		36 (0.4%)
Hodgkin lymphoma		24 (0.3%)
Brain cancer		20 (0.3%)
Other female genital cancer		13 (0.2%)
Cancer of liver		9 (0.1%)
Cancer of esophagus		9 (0.1%)
Cancer of gallbladder/bile duct		8 (0.1%)
Cancer of bone and cartilage		7(0.1%)
Other cancers in women		231 (2.8%)

## APPENDIX 3 Relative rates of CVD death in survivors of cancer after excluding those who developed cancer during the study follow up: sensitivity analysis

### 3.1

**Table:**

	Men and women *N* = 420,757 Ref: people without cancer	Men *N* = 237,670 Ref: Men without cancer	Women *N* = 183,087 Ref: Women without cancer
	Hazard ratio (95% CI)	Hazard ratio (95% CI)	Hazard ratio (95% CI)
Age‐adjusted	1.33**(1.15–1.54)	1.19(0.98–1.45)	1.53**(1.23–1.90)
Multivariable	1.39**(1.20–1.61)	1.30**(1.07–1.59)	1.55**(1.25–1.93)
Years with cancer prior to index date			
2 years ‐ 5 years	1.46**(1.16–1.85)	1.23(0.89–1.69)	1.98**(1.40–2.79)
≥ 5 years	1.35**(1.13–1.62)	1.35*(1.06–1.72)	1.37*(1.04–1.80)
Year of cancer diagnosis			
1995–2005	1.43**(1.21–1.70)	1.40**(1.11–1.76)	1.49**(1.16–1.93)
2006–2016	1.29(0.99–1.69)	1.11(0.78–1.59)	1.72**(1.15–2.56)
Cancer types			
Breast cancer	1.73**(1.29–2.34)		1.68**(1.25–2.27)
Prostate cancer		0.92(0.66–1.27)	
Melanoma	1.83**(1.33–2.53)	2.22**(1.54–3.20)	1.15(0.57–2.32)
Colorectal cancer	0.93(0.56–1.55)	0.50(0.21–1.21)	1.66(0.89–3.11)
Testicular cancer		1.08(0.27–4.31)	
Non‐Hodgkin lymphoma	1.99(0.89–4.45)	1.73(0.56–5.37)	2.26(0.73–7.01)
Kidney cancer	1.47(0.55–3.92)	1.65(0.53–5.12)	1.13(0.16–8.07)
Leukemia	1.84(0.69–4.91)	2.19(0.70–6.80)	1.24(0.17–8.83)
Cancer of bladder	2.34*(1.05–5.22)	2.01(0.75–5.38)	3.77(0.94–15.13)
Lung cancer	1.02(0.25–4.07)	‐	2.11(0.52–8.47)
Cancer of oral cavity	2.27(0.94–5.46)	2.99*(1.24–7.21)	‐
Thyroid cancer	0.94(0.30–2.91)	0.73(0.10–5.19)	1.17(0.29–4.68)
Uterine cancer			0.98(0.41–2.36)
Cervical cancer			0.57(0.08–4.07)
Ovarian cancer			2.67(0.86–8.31)
Vulvu‐vaginal			1.42(0.20–10.14)
Cancer of stomach	1.86(0.47–7.46)	2.66(0.66–10.67)	‐
Myeloma	6.46**(2.68–15.55)	6.21**(2.00–19.34)	7.75**(1.93–31.12)
Hodgkin lymphoma	1.89(0.27–13.44)	2.56(0.36–18.17)	‐
Brain cancer	8.98*(1.26–63.80)	‐	‐
Cancer of gallbladder/bile duct	9.31**(2.96–29.25)	‐	‐
Cancer of bonne and cartilage	13.77**(3.43–55.18)	‐	‐

*Note*: Patients who were cancer free prior to the CVD risk assessment date but developed cancer during follow‐up were excluded (25,627 patients composed of 11,413 men and 14,214 women were excluded).

**p* < 0.05

***p* < 0.01.
